# Human papillomavirus (HPV) genotype prevalence and impact of COVID-19 on the HPV prevention program in Duhok city

**DOI:** 10.1016/j.dialog.2022.100055

**Published:** 2022-10-03

**Authors:** Shameran Daniel, Avan Saeed Mohammed, Nashwan Ibrahim, Nawfal R. Hussein, Amer A. Balatay, Ibrahim A. Naqid, Chiman Kanaan Shekho, Dildar H. Musa, Zana Sidiq M. Saleem

**Affiliations:** a Department of Basic Sciences, College of Dentistry, University of Duhok, Kurdistan Region, Iraq; b College of Science, University of Duhok, Kurdistan Region, Iraq; c Department of Internal Medicine and Surgery, College of Medicine, University of Duhok, Kurdistan Region, Iraq; d Department of Biomedical Sciences, College of Medicine, University of Zakho, Kurdistan Region, Iraq; e Department of Pharmacology and Clinical Pharmacy, College of Pharmacy, University of Duhok, Kurdistan Region, Iraq; f Department of Biology, Faculty of Science, University of Zakho, Kurdistan Region, Iraq

**Keywords:** Human Papilloma Virus, HPV, Iraq, Genotype, Prevalence, Cross-sectional Study

## Abstract

**Introduction and aims:**

Human papillomavirus (HPV) is a sexually transmitted virus that can cause cervical cancer. This study aimed to investigate the prevalence of HPV infection, the prevalent HPV genotypes in women and men with recurrent genital infections, and the impact of the coronavirus disease 2019 (COVID-19) pandemic on the HPV prevention program.

**Materials and methods:**

This cross-sectional study was conducted in Duhok city, in the Kurdistan Region of Iraq, between January 2018 and September 2020. We recruited patients from an infectious disease clinic, who were married, were older than 18 years, and agreed to participate in this study. A reverse hybridisation-based assay was used to identify the HPV genotypes prevalent in these patients.

**Results:**

Among the patients in the study, 20.9% (67/320) tested positive for HPV infection. The HPV prevalence in females was 18.8% (52/276), which was lower than that in males (34.1%,15/44) (p = 0.21). Thirty-six patients (11.3%) were infected with a single HPV genotype, seventeen (5.3%) with two HPV genotypes, eight (2.5%) with three HPV genotypes, and the remaining six (1.8%) with four or more HPV genotypes. The most common genotypes detected among the patients were HPV-6 (7.2%), −11 (3.8%), and −16 (3.4%). The prevalence of all HPV genotype infections was highest and lowest in the 18–25- and 36–45-year age groups, respectively (*X*^*2*^ = 8.24; p = 0.041). The number of referred patients substantially reduced from 12 patients per month to 1 patient per month during the COVID-19 pandemic.

**Conclusion:**

HPV infection was common in the study population. The most common genotypes were HPV 6, 11, and 16, against which vaccines are available. Further population-based studies are needed to investigate the prevalence of such an infection.

## Highlights

• HPV infection was common in the study population from Duhak city.
• Vaccines are available for the most prevalent genotypes (HPV-6, -11, and -16).
• COVID-19 pandemic negatively impacted the HPV prevention program in Duhak city.

## 1 Introduction

Human papillomavirus (HPV) can cause oncogenic infections in different parts of the body, particularly in the genital region and cervix, and leads to cervical cancer development [1]. HPV infection is one of the most common sexually transmitted diseases worldwide, and the virus infects up to 80% of sexually active individuals at least once during their lives [1,2]. Among the 100 HPV genotypes, more than 40 HPV genotypes can cause infection in humans. HPV genotypes can be classified according to their association with cancer as follows: high-risk (HR), probable high-risk (pHR), and low-risk (LR) genotypes [1,2]. Although cervical cancer is rare in the Kurdistan Region of Iraq, recent data have shown a significant increase in cervical cancer incidence from 0.6 per 100 000 individuals in 2010 to 2 per 100 000 individuals in 2016 [[3], [4], [5]]. However, data on the prevalence of HPV in the Kurdistan Region are limited, and only a few studies have investigated the prevalence of the different HPV genotypes. For instance, a previous study of women with recurrent pelvic inflammatory diseases reported that HPV was prevalent in approximately 12% of these women, 30.7% of whom were infected with HPV genotype 16, and the majority of them were infected with more than one genotype [6]. Furthermore, previous studies have shown a lack of basic knowledge regarding HPV infection, mode of transmission, and consequences, among the general population [7]. Vaccines play a major role in controlling infection and subsequent HPV-related cancers. However, these vaccines are not a part of the vaccination program in Iraq [6]. Hence, an ambitious project was initiated in the Kurdistan Region of Iraq in 2018 to combat HPV infection. One part of the project involved studying the prevalence of the infection and the prevalent genotypes and implementing vaccine programs accordingly [6]. However, the coronavirus disease 2019 (COVID-19) pandemic, which started in March 2020 [[8], [9], [10], [11]], has negatively impacted health services in various departments [[12], [13], [14], [15]]. Recurrent genital infection is defined as three or more episodes of infection in 12 months [16,17]. Others defined recurrences genital infection as ≥4 episodes of infection within one calendar year [18]. In our study, recurrent genital infection was defined as three or more episodes of infection within 12 months. Recurrent genital infection is not uncommon and this difficult-to-manage condition affects 5–9% of women of reproductive age [19,20]. The data about such a condition is sparse in our region. One study estimated that recurrent genital infection occurs in about 5% of women of reproductive age [21]. It was previously found that patient with genital infection is at high risk to contract other sexually transmitted diseases including HPV infection [22,23]. In addition, it was previously found that recurrent vaginal infection was associated with increased odds for prevalent and incident HPV as well as delayed clearance [24,25]. This study aimed to investigate the prevalence of HPV infection in women and men with recurrent genital infections and the prevalent HPV genotype (s) among them. We then studied the impact of the COVID-19 pandemic on the HPV prevention project.

## 2 Materials and methods

### 2.1 Patient selection

This cross-sectional study was conducted between January 2018 and September 2020. We recruited patients with recurrent genital infections who did not respond to treatment and were referred to the infectious disease unit at Azadi Teaching Hospital, Duhok city, Kurdistan Region of Iraq. Since HPV infection is a sensitive subject with social and religious implications in a conservative society, we only included patients who were married and older than 18 years. In this study, we recruited 320 patients including 276 females and 44 males. The age of recruited sample ranged between 18 and 75 years old. We recruited patients agreed to participate in the study. Patients who did not give consent were excluded from the study.

In our HPV prevention program, between January 2018 and March 2020, we received more than 12 patients per month. Subsequently, due to the COVID-19 pandemic, this number dropped to approximately one patient per month.

### 2.2 Sample collection

Cervical swab samples were collected from the female patients using a cervix brush. The swab samples were then dipped into 20 mL of the transport medium (CytecCorp, Boxborough, MA, USA), according to the manufacturer’s instructions. The samples were stored at -80°C until analysis. We used penile and urethral brushing to collect samples from the male patients.

### 2.3 DNA extraction and HPV genotyping

DNA was extracted from a 400 μL aliquot of the samples incubated with proteinase K using the Qiagen Blood & Cell Culture DNA Mini Kit, according to the manufacturer’s instructions. A line probe assay (INNO-LiPA HPV Genotyping Extra II, Fujirebio, Japan), based on reverse hybridisation, was used to identify the HPV genotypes from the DNA samples. This assay detects 32 HPV genotypes simultaneously using a precise 65-base pair of polymerase chain reaction products and, hence, allows for the detection of multiple genotypes in a single sample. Internal control lines were used to confirm and detect the presence of HPV genotypes. Other controls were used to monitor sample processing (hDNA line and conjugate control line). The assay detects high risk genotypes (including 16; 18; 31; 33; 35; 39; 45; 51; 52; 56; 58; 59 and 68); Probable high-risk genotypes (including 26; 53; 66; 70; 73 and 82) and low risk genotypes (including 6; 11; 40; 42; 43; 44; 54; 61; 62; 67; 81; 83 and 89).

### 2.4 Statistical analysis

The prevalence of HPV (or its genotypes) was calculated as the percentage of all samples that were positive for HPV (or its genotypes). All data analyses were performed in an Excel spreadsheet. The Mann–Whitney test was used to compare the age of the different groups. Differences were considered statistically significant at p ≤0.05. Graphic Prism 8 (USA; San Diego; California) software was utilized to calculate the statistics.

### 2.5 Ethical approval

The study was approved by the Scientific and Ethics Committee of the College of Medicine, University of Zakho (UoZEC 2021/76). Written informed consent was obtained from all patients before the study.

## 3 Results

### 3.1 HPV prevalence and genotyping

A total of 320 outpatients (276 female and 44 male patients; 18–75 years old) were recruited during the study period. Among them, 20.9% (67/320) tested positive for one or more HPV genotypes, and the remaining 79.1% (253/320) tested negative. HPV was prevalent in 18.8% (52/276) of the female patients and less prevalent 34.1% (15/44) among the male patients (p = 0.21).

### 3.2 HPV-infection types

A total of 123 HPV genotypes were detected in 67 patients (Table 1). Among the 320 recruited patients, thirty-six patients (11.3%) were infected with a single HPV genotype, seventeen (5.3%) with two HPV genotypes, eight (2.5%) with three HPV genotypes, and the remaining six (1.8%) with four or more HPV genotypes. Among the female patients, those infected with a single HPV genotype (30, 10.9%) accounted for the highest proportion, followed by those infected with two (12, 4.3%), three (6, 2.2%), and four to six HPV genotypes (4, 1.5%,). Among the male patients, six (13.6%) and five (11.4%) patients were infected with one and two HPV genotypes, respectively, and the remaining four (9.1%) were infected with three or four HPV genotypes.

**Table 1:** Distribution and prevalence of HPV genotypes among the female and male patients in the study.

HPV Genotype	No. (%) Total patients	No. (%) Female patients	no. (%) Male patients
16	11 (3.4%)	10 (3.6%)	1 (2.3%)
18	5 (1.6%)	4 (1.5%)	1 (2.3%)
31	2 (0.6%)	1 (0.4%)	1 (2.3%)
35	1 (0.3%)	0 (0.0%)	1 (2.3%)
39	5 (1.6%)	5 (1.8%)	0 (0.0%)
45	2 (0.6%)	2 (0.7%)	0 (0.0%)
51	2 (0.6%)	2 (0.7%)	0 (0.0%)
52	3 (0.9%)	3 (1.1%)	0 (0.0%)
56	3 (0.9%)	3 (1.1%)	0 (0.0%)
58	2 (0.6%)	2 (0.7%)	0 (0.0%)
59	1 (0.3%)	1 (0.4%)	0 (0.0%)
68	2 (0.6%)	2 (0.7%)	0 (0.0%)
53	9 (2.8%)	5 (1.8%)	4 (9.1%)
66	2 (0.6%)	2 (0.7%)	0 (0.0%)
67	4 (1.3%)	2 (0.7%)	2 (4.5%)
70	1 (0.3%)	1 (0.4%)	0 (0.0%)
73	3 (0.9%)	3 (1.1%)	0 (0.0%)
82	7 (2.2%)	6 (2.2%)	1 (2.3%)
6	23 (7.2%)	12 (4.3%)	11 (25%)
11	12 (3.8%)	10 (3.6%)	2 (4.5%)
41	1 (0.3%)	0 (0.0%)	1 (2.3%)
42	2 (0.6%)	1 (0.4%)	1 (2.3%)
44	1 (0.3%)	1 (0.4%)	0 (0.0%)
54	6 (1.9%)	5 (1.8%)	1 (2.3%)
61	2 (0.6%)	2 (0.7%)	0 (0.0%)
62	2 (0.6%)	2 (0.7%)	0 (0.0%)
81	3 (0.9%)	2 (0.7%)	1 (2.3%)
84	2 (0.6%)	2 (0.7%)	0 (0.0%)
87	4 (1.3%)	2 (0.7%)	2 (4.5%)

### 3.3 Distribution and prevalence of HPV genotypes per female and male patient

The HPV genotypes found in our patients included HR-HPV-16, −18, −31, −35, −39, −45, −51, −52, −56, −58, −59, and −68; pHR-HPV-53, −66, −67, −70, −73, and −82; and LR-HPV-6, −11, −41, −42, −44, −54, −61, −62, −81, −84, and −87. Eleven HR-HPV genotypes (HPV-16, −18, −39, −52, −56, −45, −51, −58, −68, −31, and −59) were present among the female patients, while only four HR-HPV genotypes (HPV-16, −18, −31, and −35) were present among the male patients; six pHR-HPV genotypes (HPV-82, −53, −73, −66, −67, and −70) were detected among the female patients (HPV-82, −53, −73, −66, −67, and −70), and three pHR-HPV genotypes (HPV-53, −67, and −82) were detected among the male patients. Furthermore, eleven LR-HPV genotypes (HPV-6, −11, −54, −61, −81, −84, −62, −66, −87, −44, and −42) were present among the female patients and seven (HPV-6, −11, −87, −41, −42, 54, and −81) among the male patients (Table 2).

**Table 2:** Distribution of the detected HPV genotypes according to the risk among the female and male patients in the study.

Sex	HR-HPV	pHR-HPV	LR-HPV
Female	35 (12.7%)	19 (6.9%)	39 (14.1%)
Male	4 (9.1%)	7 (15.9%)	19 (43.2%)
Total	39 (12.2%)	26 (8.1%)	58 (18.1%)

HR: high-risk; pHR: probable high-risk; LR: low-risk.

### 3.4 Prevalence of HPV infection by patient age

HPV infection was the most prevalent in the 18–25-year age group and least in the 36–45-year age group (*X*^*2*^ = 8.24; p = 0.041) (Table 3). Among the female patients, the prevalence of HPV infection was 54.3% (19/35), 34.7% (43/124), 24.7% (18/73), and 29.5% (13/44) (*X*^*2*^ = 9.7; p = 0.021) in the 18–25-, 25–35-, 36–45-, and ≥46-year age groups, respectively. Unlike with the female patients, the prevalence of HPV infection among the male patients peaked in the 26–35-year group (87.5%, 14/16,) and ≥46-year age group (75%, 6/8). The difference in prevalence among the different age groups in men was not significant (*X*^*2*^ = 5.97; p = 0.11).

**Table 3:** Prevalence of HPV infection by age among the female and male patients in the study.

	Female	Male	Total
18–25 years old	54.3% (19/35)	50% (4/8)	53.5% (23/43)
26–35 years old	34.7% (43/124)	87.5% (14/16)	40.7% (57/140)
36–45 years old	24.7% (18/73)	50% (6/12)	28.2% (24/85)
≥ 46 years old	29.5% (13/44)	75% (6/8)	36.5% (19/52)

## 4 Discussion

Cervical cancer is a preventable disease [26]. It can be prevented through the implementation of screening and vaccination program. The data about the prevalence of HPV infection is sparse in the region therefore we aimed to provide information about the prevalence of HPV infection, the prevalent HPV genotypes in women and men with recurrent genital infections. Social and religious barriers surrounding the subject of HPV [6] make it extremely difficult to conduct a population-based study. Therefore, we recruited patients with recurrent genital infections in our study. To the best of our knowledge, our study is the first to recruit both male and female patients. In our study, in female patients, the prevalence rates of HPV and HR-HPV were 20.9% and 12.2%, respectively, and in women, these rates were 18.8% and 12.7%, respectively. In a study conducted in Turkey, the prevalence of HPV was reported between 2.0% and 46.0% in some regional studies [27,28]. A study conducted in Qatar recruiting female patients reported an HPV DNA prevalence of 8.1% [29]. The prevalence rates of HPV and HR-HPV reported by a study conducted in Iran were 14.4% and 6.5%, respectively, and another study conducted in China reported these rates to be 22.7% and 17.3%, respectively [30]. Studies conducted in Portugal and France found HPV prevalence rates of 19% and 15%, respectively [31,32]. Furthermore, the prevalence rates of HPV and HR-HPV reported by a study conducted in USA were 40% and 20.4%, respectively [33]. The discrepancies in the findings among different countries can be explained by differences in the sampling method, sample size, techniques used for the detection of HPV, and social and racial differences.

In our study, the prevalence rates of HPV and HR-HPV in men were 34.1% and 9.1%, respectively. Previous studies have shown great variation in the prevalence of HPV infection in men. The prevalence depended on the recruitment of patients for the study. In a previous study, the prevalence of HPV was studied in high risk men, and the HPV prevalence was found to reach up to 93% [34] while the prevalence was 72% when asymptomatic males were recruited [35]. In a study conducted in the USA, the prevalence rates of HPV and HR-HPV among men were 45.2% and 25.1%, respectively [36]. Owing to the small sample size of our study, more studies are needed to increase the sample size.

In agreement with previous studies [[37], [38], [39]], among the HR genotypes, HPV genotype 16 was the most common genotype detected in our study, whereas the most common LR genotype was HPV genotype 6. Our results agree with previous studies conducted in Turkey where HPV type 16 and type 18 prevalence was reported to be the most common HPV genotypes [40]. Besides, studies conducted in Iran showed that HPV 16 was the most common detected virus genotype [[41], [42], [43]]. In addition, in a study conducted in Saudi Arabia, HPV genotypes 16; 18 and 45 are the most common genotypes in Saudi Arabia and are responsible for about 70% of all cervical cancer cases in the country [44].

A previous study found two peaks of HPV prevalence in females aged 26–30 and 46–50 years, respectively [45]. In our study, HPV prevalence peaked in females aged 18–25 years. There was a slight increase in the prevalence of HPV infections in women older than 45 years. This slight increase might be attributed to the increase in screening rates for older women. The age pattern of HPV prevalence found in our study was in agreement with that in a study conducted in Ghana, where the prevalence peaked in women around 20 years old and older than 45 years [46]. This U-shaped pattern of age distribution may not reflect the reality and might be attributed to increased screening rates in these two age groups because women at an early stage of marriage and older women seek more medical advice. In agreement with another study investigating the age distribution pattern of HPV infection in men [36], we found a two-peak pattern among the male patients in our study. The first peak was in the 26–35-year age group and second in the ≥46-year age group. A larger sample size is needed to confirm our results and explain the pattern of distribution.

This study was a part of the HPV prevention program and was performed to collect baseline information about HPV infection to implement a region-wide prevention program. However, the number of patients we were able to recruit substantially reduced owing to the COVID-19 pandemic. As most resources were diverted towards combating COVID-19, we faced limitations in funding and other necessary resources to continue our project. An urgent plan is required to provide funds and resources for resuming the project. Stopping such programs may have disastrous consequences in the future.

The major strengths of our study are that we examined HPV genotypes in both male and female patients and in a relatively large sample size. Our results have clinical implications as we showed that HPV infection is not uncommon and urgent plan is needed for screening and vaccination. Our study has several limitations. Our sampling method was not random. Furthermore, HPV infection can be asymptomatic, but we recruited only patients with recurrent infections. Therefore, this study cannot be considered a prevalence study for this region. However, performing community-based studies in this society is extremely difficult because of the stigma attached to HPV infection. The government should encourage all women to undergo regular screening. Owing to social stigma, recruiting male participants was also difficult; hence, there was a bias in our sample collection.

In conclusion, HPV infection is not uncommon in Duhok city. The most common genotypes were HPV-6, -11, and -16, which are covered by the available vaccines. However, the COVID-19 pandemic adversely impacted the HPV prevention program and led to its closure.

## Funding

This research did not receive any specific grant from funding agencies in the public, commercial, or not-for-profit sectors.

## Declaration of Competing Interest

The authors declare that they have no known competing financial interests or personal relationships that could have appeared to influence the work reported in this paper.

## References

[1] Brianti P., De Flammineis E., Mercuri S.R. (2017). Review of HPV-related diseases and cancers. New Microbiol.

[2] Kombe Kombe A.J., Li B., Zahid A., Mengist H.M., Bounda G.-A., Zhou Y. (2021). Epidemiology and burden of human papillomavirus and related diseases, molecular pathogenesis, and vaccine evaluation. Front Public Health.

[3] Othman R.T., Abdulljabar R., Saeed A., Sadiq S., Kittani H.M., Mohammed S.A. (2011). Cancer Incidence Rates in the Kurdistan Region/Iraq from. Asian Pac J Cancer Prev.

[4] Kocarnik J.M., Compton K., Dean F.E., Fu W., Gaw B.L., Harvey J.D. (2022). Cancer incidence, mortality, years of life lost, years lived with disability, and disability-adjusted life years for 29 cancer groups from 2010 to 2019: a systematic analysis for the Global Burden of Disease Study 2019. JAMA Oncol.

[5] Tran K.B., Lang J.J., Compton K., Xu R., Acheson A.R., Henrikson H.J. (2022). The global burden of cancer attributable to risk factors, 2010–19: a systematic analysis for the Global Burden of Disease Study 2019. The Lancet.

[6] Hussein N.R., Balatay A.A., Assafi M.S., AlMufty T.A. (2016). High risk human papilloma virus genotypes in Kurdistan region in patients with vaginal discharge. Asian Pac J Cancer Prev.

[7] Ibrahim W.A., Daniel S., Hussein N.R., Assafi M.S., Othman R. (2019). Knowledge of Human Papillomavirus (HPV) and the HPV Vaccine Among Medical and Nursing Students of Duhok, Iraq. Women’s Health Bulletin.

[8] Hussein N.R. (2020). The Role of Self-Responsible Response Versus Lockdown Approach in Controlling COVID-19 Pandemic in Kurdistan Region of Iraq. Int J Infect.

[9] Hussein N.R., Naqid I.A., Jacksi K., Abdi B.A. (2020). Assessment of knowledge, attitudes, and practices toward COVID-19 virus among university students in Kurdistan region, Iraq: Online cross-sectional study. J Family Med Primary Care.

[10] Hussein N.R., Naqid I.A., Saleem Z.S.M. (2020). A retrospective descriptive study characterizing coronavirus disease epidemiology among people in the Kurdistan Region, Iraq. Medit J Hematol Infect Dis.

[11] Hussein N.R., Naqid I.A., Saleem Z.S.M., Almizori L.A., Musa D.H., Ibrahim N. (2020). A sharp increase in the number of COVID-19 cases and case fatality rates after lifting the lockdown in Kurdistan region of Iraq. Ann Med Surgery.

[12] Hussein N. (2020). The Impact of COVID-19 Pandemic on the Elimination of Viral Hepatitis in Duhok City, Kurdistan Region of Iraq. Hepatitis Monthly.

[13] Hussein N.R., Daniel S., Mirkhan S.A., Saleem Z.S.M., Musa D.H., Ibrahim N. (2020). Impact of the Covid-19 pandemic on the elimination of hepatitis C virus in Duhok, Kurdistan, Iraq: A retrospective cross-sectional study. J Family Med Primary Care.

[14] Hussein N.R., Musa D.H., Ibrahim N., Naqid I.A., Saleem Z.S.M., Jacksi K. (2020). Impact of Covid-19 pandemic on surgical practice in Kurdistan, Iraq: An online cross-sectional survey. Int J Surgery Open.

[15] Hussein N.R., Saleem Z.S.M., Ibrahim N., Musa D.H., Naqid I.A. (2020). The impact of COVID-19 pandemic on the care of patients with kidney diseases in Duhok City, Kurdistan Region of Iraq. Diabetes Metab Syndr Clin Res Rev.

[16] Hay P., Ugwumadu A., Chowns J. (1997). Sex, thrush and bacterial vaginosis. Int J STD AIDS.

[17] Cook R.L., Redondo-Lopez V., Schmitt C., Meriwether C., Sobel J.D. (1992). Clinical, microbiological, and biochemical factors in recurrent bacterial vaginosis. J Clin Microbiol.

[18] Lema V.M. (2017). Recurrent vulvo-vaginal candidiasis: diagnostic and management challenges in a developing country context. Obstetrics Gynecol Int J.

[19] Lines A., Vardi-Flynn I., Searle C. (2020). Recurrent vulvovaginal candidiasis. BMJ.

[20] Matheson A., Mazza D. (2017). Recurrent vulvovaginal candidiasis: A review of guideline recommendations. Aust N Z J Obstet Gynaecol.

[21] MohammedAB A.J., Abdullah S. (2015). Identification of Candida spp. isolated from vaginal swab by phenotyp. ic methods and multiplex PCR in Duhok, Iraq. Int J Res Med Sci.

[22] Johnson A.M., Mercer C.H., Beddows S., de Silva N., Desai S., Howell-Jones R. (2012). Epidemiology of, and behavioural risk factors for, sexually transmitted human papillomavirus infection in men and women in Britain. Sex Transm Infect.

[23] Haydar S.O., Naqid I. (2022). A Study of Bacterial Vaginosis and Associated Risk Factors among Married Women in Zakho City, Kurdistan Region, Iraq. J Life Bio Sci Res.

[24] King C.C., Jamieson D.J., Wiener J., Cu-Uvin S., Klein R.S., Rompalo A.M. (2011). Bacterial Vaginosis and the Natural History of Human Papillomavirus. Infect Dis Obstet Gynecol.

[25] Vieira-Baptista P., Lima-Silva J., Pinto C., Saldanha C., Beires J., Martinez-de-Oliveira J. (2016). Bacterial vaginosis, aerobic vaginitis, vaginal inflammation and major Pap smear abnormalities. Eur J Clin Microbiol Infect Dis.

[26] Galles N.C., Liu P.Y., Updike R.L., Fullman N., Nguyen J., Rolfe S. (2021). Measuring routine childhood vaccination coverage in 204 countries and territories, 1980–2019: a systematic analysis for the Global Burden of Disease Study 2020, Release 1. The Lancet.

[27] Ozcelik B., Serin I., Basbug M., Erez R. (2003). Human papillomavirus frequency of women at low risk of developing cervical cancer: a preliminary study from a Turkish university hospital. Eur J Gynaecol Oncol.

[28] Hancer V.S., Buyukdogan M., Bylykbashi I., Oksuz B., Acar M. (Oct-Dec 2018). Prevalence of Human Papilloma Virus Types in Turkish and Albanian Women. J Cytol.

[29] Elmi A.A., Bansal D., Acharya A., Skariah S., Dargham S.R., Abu-Raddad L.J. (2017). Human Papillomavirus (HPV) Infection: Molecular Epidemiology, Genotyping, Seroprevalence and Associated Risk Factors among Arab Women in Qatar. PLoS One.

[30] Wu X., Zhao J., Cui X., Li Q., Tao H., Pan Q. (2017). Prevalence of type-specific human papillomavirus infection among 18-45 year-old women from the general population in Liuzhou, Guangxi Zhuang Autonomous Region: a cross-sectional study. Zhonghua liu xing bing xue za zhi.

[31] Pista A., de Oliveira C.F., Cunha M.J., Paixao M.T., Real O., C. P. S. Group (2011). Prevalence of human papillomavirus infection in women in Portugal: the CLEOPATRE Portugal study. Int J Gynecol Cancer.

[32] Monsonego J., Zerat L., Syrjänen K., Zerat J.-C., Smith J.S., Halfon P. (2012). Prevalence of type-specific human papillomavirus infection among women in France: Implications for screening, vaccination, and a future generation of multivalent HPV vaccines. Vaccine.

[33] McQuillan G.M., Kruszon-Moran D., Markowitz L.E., Unger E.R., Paulose-Ram R. (2017).

[34] Olesen T.B., Sand F.L., Rasmussen C.L., Albieri V., Toft B.G., Norrild B. (2019). Prevalence of human papillomavirus DNA and p16<sup>INK4a</sup> in penile cancer and penile intraepithelial neoplasia: a systematic review and meta-analysis. Lancet Oncol.

[35] Smith J.S., Gilbert P.A., Melendy A., Rana R.K., Pimenta J.M. (2011). Age-specific prevalence of human papillomavirus infection in males: a global review. J Adolesc Health.

[36] Han J.J., Beltran T.H., Song J.W., Klaric J., Choi Y.S. (2017). Prevalence of Genital Human Papillomavirus Infection and Human Papillomavirus Vaccination Rates Among US Adult Men: National Health and Nutrition Examination Survey (NHANES) 2013-2014. JAMA Oncol.

[37] Wang J., Tang D., Wang K., Wang J., Zhang Z., Chen Y. (2019). HPV genotype prevalence and distribution during 2009-2018 in Xinjiang, China: baseline surveys prior to mass HPV vaccination. BMC Womens Health.

[38] Jiang L., Tian X., Peng D., Zhang L., Xie F., Bi C. (2019). HPV prevalence and genotype distribution among women in Shandong Province, China: Analysis of 94,489 HPV genotyping results from Shandong's largest independent pathology laboratory. PLoS One.

[39] Bitarafan F., Hekmat M.R., Khodaeian M., Razmara E., Ashrafganjoei T., Modares Gilani M. (2021). Prevalence and Genotype Distribution of Human Papillomavirus Infection among 12 076 Iranian Women. Int J Infect Dis.

[40] Dursun P., Ayhan A., Mutlu L., Çağlar M., Haberal A., Güngör T. (2013). HPV types in Turkey: multicenter hospital based evaluation of 6388 patients in Turkish gynecologic oncology group centers. Turk Patoloji Derg.

[41] Jamdar F., Farzaneh F., Navidpour F., Younesi S., Balvayeh P., Hosseini M. (2018). Prevalence of human papillomavirus infection among Iranian women using COBAS HPV DNA testing. Infect Agents Cancer.

[42] Wu E.-Q., Liu B., Cui J.-F., Chen W., Wang J.-B., Lu L. (2013). Prevalence of type-specific human papillomavirus and pap results in Chinese women: a multi-center, population-based cross-sectional study. Cancer Causes Control.

[43] Sjoeborg K.D., Tropé A., Lie A.K., Jonassen C.M., Steinbakk M., Hansen M. (2010). HPV genotype distribution according to severity of cervical neoplasia. Gynecol Oncol.

[44] Mousa M., Al-Amri S.S., Degnah A.A., Tolah A.M., Abduljabbar H.H., Oraif A.M. (Nov-Dec 2019). Prevalence of human papillomavirus in Jeddah, Saudi Arabia. Ann Saudi Med.

[45] Chan P.K., Chang A.R., Yu M.Y., Li W.H., Chan M.Y., Yeung A.C. (Jan 1 2010). Age distribution of human papillomavirus infection and cervical neoplasia reflects caveats of cervical screening policies. Int J Cancer.

[46] Awua A.K., Adanu R.M.K., Wiredu E.K., Afari E.A., Severini A. (2017). Differences in age-specific HPV prevalence between self-collected and health personnel collected specimen in a cross-sectional study in Ghana. Infect Agents Cancer.

